# Augmentation strategies for an imbalanced learning problem on a novel COVID-19 severity dataset

**DOI:** 10.1038/s41598-023-45532-2

**Published:** 2023-10-25

**Authors:** Daniel Schaudt, Reinhold von Schwerin, Alexander Hafner, Pascal Riedel, Manfred Reichert, Marianne von Schwerin, Meinrad Beer, Christopher Kloth

**Affiliations:** 1 Department of Computer Science, Ulm University of Applied Science, Albert-Einstein-Allee 55, 89081 Ulm, Baden-Wurttemberg Germany; 2 Institute of Databases and Information Systems, Ulm University, James-Franck-Ring, 89081 Ulm, Baden-Wurttemberg Germany; 3 Department of Radiology, University Hospital of Ulm, Albert-Einstein-Allee 23, 89081 Ulm, Baden-Wurttemberg Germany

**Keywords:** Machine learning, Predictive medicine, Medical imaging

## Abstract

Since the beginning of the COVID-19 pandemic, many different machine learning models have been developed to detect and verify COVID-19 pneumonia based on chest X-ray images. Although promising, binary models have only limited implications for medical treatment, whereas the prediction of disease severity suggests more suitable and specific treatment options. In this study, we publish severity scores for the 2358 COVID-19 positive images in the COVIDx8B dataset, creating one of the largest collections of publicly available COVID-19 severity data. Furthermore, we train and evaluate deep learning models on the newly created dataset to provide a first benchmark for the severity classification task. One of the main challenges of this dataset is the skewed class distribution, resulting in undesirable model performance for the most severe cases. We therefore propose and examine different augmentation strategies, specifically targeting majority and minority classes. Our augmentation strategies show significant improvements in precision and recall values for the rare and most severe cases. While the models might not yet fulfill medical requirements, they serve as an appropriate starting point for further research with the proposed dataset to optimize clinical resource allocation and treatment.

## Introduction

Screening infected patients with fast and reliable methods is a key learning from the COVID-19 pandemic. Developing machine learning models to assist clinical decision making in the beginning of a pandemic can be critical as it can shorten time-to-diagnosis and support specialized medical staff in an emergency setting^1^. Patients with severe COVID-19 show rapid progression with respiratory failure, respiratory distress syndrome, septic shock or even death within a short period of time^2^. The likelihood of necessary intubation is higher with greater severity, rendering the severity valuable clinical information to assess and to allocate critical hospital capacity. It is therefore essential to not only diagnose COVID-19 but also predict disease severity, especially to support medical staff in an emergency setting.

The analysis of chest X-ray images (CXR) can be a promising approach to predict severity, especially because the testing via real-time polymerase chain reaction (RT-PCR) is not conclusive for disease severity. Diagnosis on X-ray images is more widely used, shows a larger availability and safer use to control the spread of the virus when compared with computer tomography^3^.

Deep learning models require large amounts of data^4–6^ to train and although large publicly available COVID-19 CXR datasets exist by now, many do not include indication of disease severity. This makes the development of appropriate models difficult. In this work we publish severity labels for the 2358 COVID-19 positive images in the COVIDx8B dataset^7,8^, creating one of the largest collections of publicly available COVID-19 severity data. The proposed severity scores range from 1 (mild) to 5 (critical) and have been verified and labeled by a dedicated thoracic radiologist (C.K.) with 9 years of experience in lung imaging.

**Figure 1:**
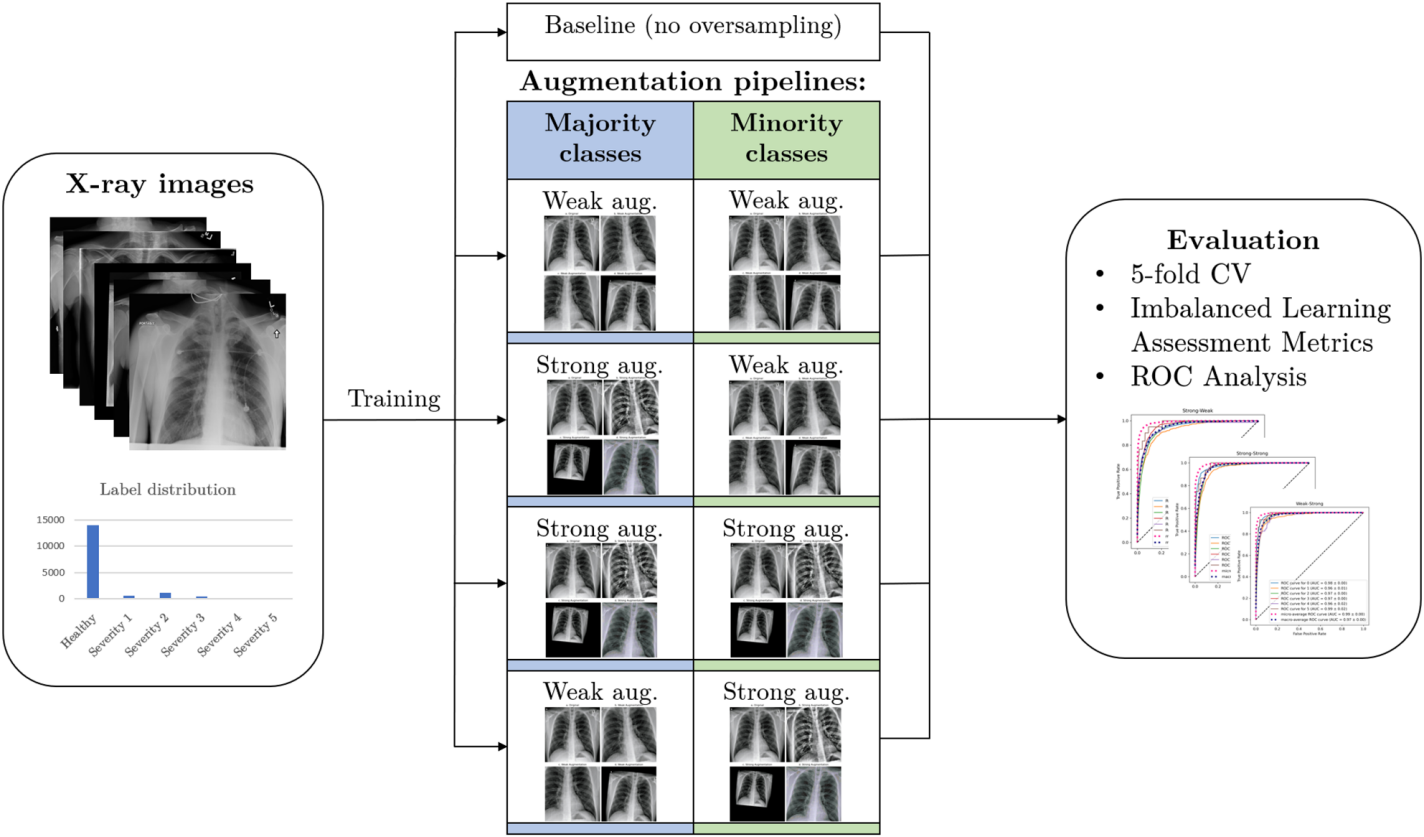
Schematic representation of the research problem of this paper and the proposed augmentation strategies.

Building on this dataset, we train and evaluate deep learning models to provide a first benchmark for the severity classification task. Since the distribution of severity scores naturally follows a skewed distribution, where the most severe cases are very rare, we encounter an imbalanced learning problem. This strongly hinders the performance of learning algorithms^9,10^, especially for the most severe cases, which is very much undesirable in this context. To improve classification and detection of these cases, we propose multiple augmentation strategies for the majority and minority classes. We examine the effect of these strategies on appropriate evaluation metrics and note significant improvements in the respective precision and recall values. These pipelines can serve as a first indication on how to improve classification for the newly created dataset. Figure 1 shows a schematic representation of the research problem of this paper and the proposed augmentation strategies. The data and code from this study is available under https://github.com/dschaudt42/covid-severity-aug.

The main contributions of our work are:

- We provide severity scores from 1 to 5 for all COVID-19 positive images in the COVIDx8B CXR data collection, making it one of the largest COVID-19 severity databases with 2358 labeled CXR images.
- We train and evaluate deep learning models on the newly created dataset to provide a benchmark for the severity classification task.
- We identify the imbalanced class distribution for severity classes as a major challenge for this use-case and propose multiple augmentation strategies to alleviate this problem. Our augmentations are class-specific and improve the classification of the most severe and underrepresented cases.

Targeting less frequent classes with specific augmentations is so far an underexplored research area. Although it is common to synthesize new samples for minority classes with sampling methods^11–13^ or generative models^14,15^, we do not see the same rigorous research towards class-specific augmentation strategies. We aim to somewhat close this gap and initiate the discussion in this area.

## Related work

### COVID-19 severity

There exist many works applying deep learning to CXR images to detect a COVID-19 pulmonary disease^7,16–19^ or pneumonia in general^20–22^. However, not as many studies integrate disease severity, mostly because suitable data can be limited or costly. Some notable work regarding severity prediction with various machine learning models has been done on tabular data (clinical data, demographic data, etc.)^23–27^ or image data^28–38^ or a combination of both^39–41^.

Schöning et al.^26^ use demographic data, medical history, and laboratory values to train machine learning models to predict severe and non-severe cases. Similarly, Quiroz et al.^27^ use a combination of clinical and imaging features to predict whether a patient diagnosed with COVID-19 is likely to have mild or severe disease. They also encounter a highly imbalanced dataset and examine 4 different oversampling techniques. Alballa and Al-Turaiki^42^ give an overview of COVID-19 severity prediction based on structured data for classical machine learning models.

Lassau et al.^41^, Chieregato et al.^39^, and Ho et al.^40^ combine features extracted from computed tomography (CT) images and clinical data to predict severity outcomes. Signoroni et al.^43^ propose a multi-network architecture in an end-to-end scheme to segment, align, and predict COVID-19 severity, while also publishing a large severity dataset with 4695 images. Danielov et al.^28^ use a multi-stage process consisting of lung segmentation and disease segmentation to predict a severity score based on the percentage of covered lung segments in X-ray images. Qiblawey et al.^29^ employ a similar approach based on CT images, predicting mild, moderate, severe, and critical cases. Shan et al.^44^ use a support vector machine to predict severity based on extracted mass of infection values for 5 lung lobes on CT images. La Salvia et al.^30^ predict COVID-19 severity based on lung ultrasound images using two severity scales.

Sayed et al.^31^ use a combination of convolutional neural network (CNN) extracted features and spatial and frequency based handcrafted features from X-ray images to predict COVID-19 severity with six different classifiers. Zandehshahvar et al. predict COVID-19 severity in 4 classes normal, mild, moderate, and severe for X-ray images. They construct a latent space representation of their model to visualize disease progression for single patients^32^. Blain et al.^33^ predict severity on a scale from 0 to 3 based on alveolar and interstitial opacity on X-ray images in a multiclass deep learning framework. Cohen et al. and Wong et al. predict severity based on geographic extent scoring and opacity extent scoring with a CNN model on X-ray images. Aboutalebi et al.^38^ extend upon this area in the direction of airspace disease grading and propose a CNN for predicting the airspace severity of a COVID-19 positive patient.

### Imbalanced classification

Skewed class distributions and underrepresented data can negatively impact the performance of machine learning models^9,10^. Resampling methods like undersampling and oversampling can modify the class distribution during training to artificially decrease the level of imbalance^45^. While undersampling removes samples from the majority class, oversampling appends samples from the minority class to even the class distribution. In the most basic form the removed or added samples are picked randomly, hence the terms *random undersampling* (RUS) and *random oversampling* (ROS). More sophisticated approaches can employ metaheuristics and optimization algorithms to pick fitting samples^46,47^.

The loss of information through RUS can increase volatility in training, especially if class imbalance is very high. Therefore, ROS is prefered in most cases^48^. While the method is simple and can be applied to many domains, the repeated drawing of the same sample can lead to overfitting^49^. To counter this, more complex methods like SMOTE^11,12^ or ADASYN^13^ create synthetic samples of the minority class by interpolating between nearest neighbors. Generative adversarial networks (GANs)^50^ have also been used to create synthetic samples to increase minority classes^14,51^.

In the context of medical imaging, Wang et el. use a Wasserstein GAN to improve classification for lung nodules in CT images^52^. Schaudt et al. propose a StyleGAN^53^, trained with differentiable augmentation^54^ to improve COVID-19 detection on a small amount of lung X-ray images^55^. Saini and Susan use a Deep Convolutional GAN (DCGAN) to rebalance histopathological images for breast cancer detection^15^. Reza and Ma compare different oversampling techniques like SMOTE and ADASYN on histopathology microscopic images to predict cancerous and non-cancerous tissue^56^. Shi et al.^57^ use data augmentation to conduct a pre-finetuning step to adapt a pretrained model to have an initial representation of the target data before the training takes place. This is similar to the idea conceived in this work, with the difference of using the augmented data only in a pre-finetuning step, while we rebalance the whole training with augmented data.

## Materials and methods

In this work we provide a severity score for each COVID-19 positive image in the COVIDx8B dataset and train a deep learning model on these scores. We specifically examine different augmentation strategies to use in combination with random oversampling to improve classification of the most severe cases, which are highly underrepresented. This section describes the data and scoring, as well as the training of our model with these strategies.

### Data

The COVIDx8B dataset is currated by Wang et al. and the University of Waterloo, Canada^7,8^ and contains COVID-19 CXR images from multiple sources: RICORD^58^, Cohen et al.^59^, RSNA^60^ and the COVID-19 Radiography Database^61^. All data sources are publicly available. The COVIDx dataset was originally used to build the COVID-Net model^7^ but has since significantly grown in size. The dataset contains 16,352 CXR images coming from patients of at least 51 countries, but does not provide detailed information on patient’s demographics. Since the COVIDx8B dataset is build by extracting image from multiple sources (to avoid patient overlap), an exact patient demographic can not be given. Some source datasets provide demographic information in various details. The RICORD database has only COVID-19 positive cases from 645 male and 353 female patients, with an average age of 56 years^58^. Cohen et al. contains 559 male patients and 311 female patients with an average age of 54 years. Most of the COVID-19 negative images are extracted from RSNA database^60^.

The COVIDx8B dataset is split into training and testing subsets. The training subset contains 15,952 images, from which 2,158 are COVID-19 positive and 13,794 are COVID-19 negative. The test subset contains 200 COVID-19 positive and 200 COVID-19 negative images. For a comparison of binary classification performance on the original dataset see Breve^62^. Since we utilize cross-validation to evaluate our models, we combine both training and test subsets. In this work we provide a severity score for each COVID-19 positive image in the COVIDx8B dataset. The ethics board of the Medical Faculty and the University Hospital in Ulm approved this retrospective evaluation study and waived the informed consent requirement (No. 271/20).

#### Severity scoring

The combined training and test data contains 2358 COVID-19 positive images, which we labeled with a severity score ranging from 1 to 5. The ethics board of the Medical Faculty and the University Hospital in Ulm approved this retrospective data evaluation study and waived the informed consent requirement (No. 271/20). A dedicated thoracic radiologist (C.K.) with 9 years of experience in lung imaging verified and labeled the data. 60 images were dropped, since they presented no indication of the presence of opacities, leaving 2298 images with a severity score. Table 1 shows the distribution of labels in the final dataset. To the best of our knowledge, this facilitates one of the largest collections of severity information on COVID-19 positive CXR images.

**Table 1:** Label distribution.

Label	Count
Healthy	14054
Severity 1	566
Severity 2	1145
Severity 3	496
Severity 4	74
Severity 5	17

**Figure 2:**
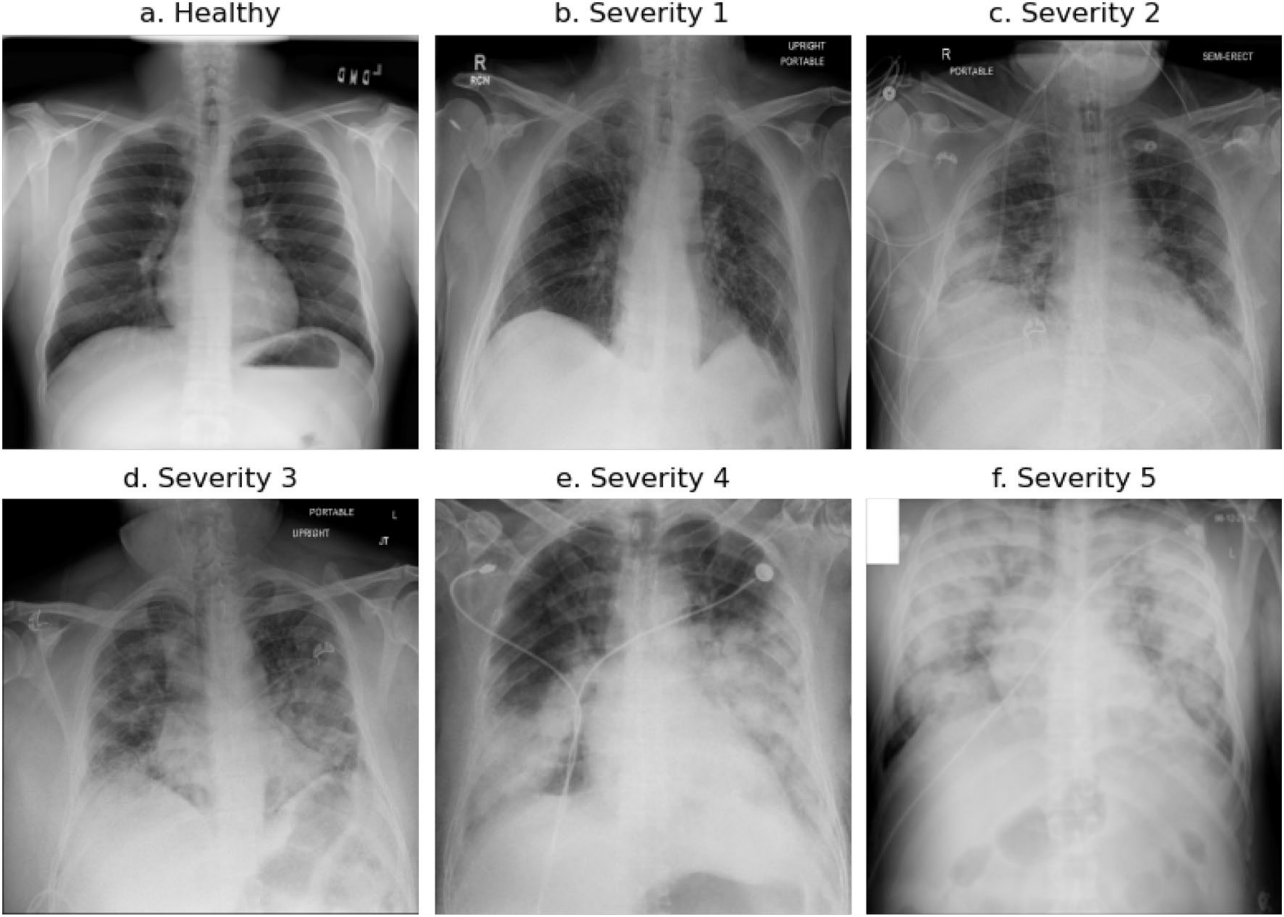
Example chest X-ray images of healthy patient (**a**) and severity scoring 1–5 (**b**–**f**).

There are some typical imaging features of COVID-19 pneumonia that can be registered both on CT and CXR images. The main findings are consolidations and hazy ground-glass opacities. The distribution is typically bilateral, however in an initial state manifestations on only one side can be registered. Especially ground-glass opacities are usually multifocal, bilateral and peripheral. Additional central manifestations can also be subdivided. If manifestations were registered on both sides, some of the lobes can be affected or all lobes (panlobar). Sometimes subpleural bands, architectural distortions, peribronchial thickening and traction bronchiectasis can be registered. The classification of the manifestation type is oriented and modified to the established multivalued Brixia score^43,63,64^. There is no quantification using an additional algorithm. Quantitative assessment of lung involvement percentages is oriented and adapted to CT imaging^65,66^. Figure 2 shows image examples for all severity scores. The severity score can be described as:

- *Healthy* No lung abnormalities.
- *Severity 1* Interstitial infiltrates, ground-glass opacities<25% of volume of the lung, no consolidations.
- *Severity 2* Interstitial and alveolar infiltrates, interstitial dominant with ground-glass opacities 25-50% of volume of the lung. Even small consolidations.
- *Severity 3* Similar interstitial and alveolar infiltrates, 50-75% of volume of the lung.
- *Severity 4* Interstitial and alveolar infiltrates, alveolar dominant, 50-75% of volume of the lung.
- *Severity 5* Acute respiratory distress syndrome (ARDS) features,>75% of volume of the lung is affected.

### Training details

Since we want to focus on the effect of our augmentation strategies, we are not overly concerned with the type or architecture of the selected model, as well as the most optimal performance. Therefore, we select a ConvNeXt-S^67^ model to carry out our experiments. These model types achieve state-of-the-art performance on a variety of image classification tasks and have been used extensively in academic literature.

All models have been pretrained on the ImageNet^68^ database. This allows us to use finely calibrated weights as a starting point for our training. Contrary to traditional transfer learning, we do not freeze any weights for the training process, but use all gradients for updates. This is to compensate for the shift in image distributions between the pretraining data and our CXR data. ImageNet comprises a diverse dataset with 1000 classes and therefore has a different image space compared to the desaturated CXR images of this study. We replace the final layer of ConvNeXt-S with a linear layer of 6 output nodes, one for each class.

The hyperparameter settings for all models are shown in Table 2. We keep these hyperparameters constant for all trained models to validate the effect of our augmentation pipelines. To make the comparison between models fair, we use the same amount of training epochs (40 each). The final model corresponds to the model with the lowest validation error after each epoch of training per cross-validation split. In our case, 40 epochs are more than enough for each model to converge. The input image size is $224\times 224$, which the model was optimized for during pretraining. All images are resized with bilinear interpolation and normalized with the mean and standard deviation values from ImageNet^68^ images. Although the image space of this study is different from ImageNet, changing these values would interfere with the pretrained models. The input tensors are of shape [batchsize,channels,height,width], resulting in input dimensions of [16,3,224,224] in our experiments. The output tensor is of shape [1,6], representing class probabilities of the 6 classes in the dataset by applying a softmax function. We use PyTorch^69^ to carry out the computations.

**Table 2:** Training settings for all models.

Hyperparameter	Value
Optimizer	Adam^70^
Loss function	Cross-entropy
Batchsize	16
Base learning rate	1e−4
Learning rate scheduler	Cosine decay
Training epochs	40
Optimizer momentum	$\beta _1,\beta _2 = 0.9,0.999$
Dropout	Classification layer p = 0.5

### Augmentation strategies for oversampling

One of the main goals for this work is to improve classification and detection of underrepresented severity classes. This is especially important because the most severe cases have the lowest occurrences. To improve classification metrics for these cases and artificially create a balanced dataset, we apply ROS. This method randomly selects samples of the minority class and feeds copies of them to the model during training. This leads to a uniform distribution of classes during training, but repeats the same images multiple times. To increase image variety of the minority classes, we present and examine specific augmentation strategies that are applied during training. We utilize these strategies with ROS, such that different augmentation pipelines are being used for the majority and minority classes. The following sections describe these strategies, pipelines and concomitant models. All augmentations are carried out with the Albumentations library^71^. This work utilizes the following augmentations:

- *ShiftScaleRotate* This augmentation randomly translates, scales and rotates an image within the specified limits and uses bilinear interpolation.
- *CLAHE* This augmentation applies Contrast Limited Adaptive Histogram Equalization (CLAHE) to improve the contrast in images. It is an adaptive histogram equalization method that limits the contrast amplification and therefore reduces overamplification of noise in homogeneous regions of an image^72^. An upper threshold for contrast limiting is set with clip_limit.
- *RandomBrightnessContrast* This augmentation randomly changes brightness and contrast of an image by applying addition and multiplication point operators respectively within the specified limits.
- *RandomGamma* This augmentation randomly adjusts gamma within the specified limits.
- *Sharpen* This augmentation sharpens an image and overlays the result with the original image by applying a convolution between a sharpening kernel and the image.
- *Blur* This augmentation blurs an image using a random-sized, normalized kernel within specified limits to average pixel values.
- *MotionBlur* This augmentation blurs an image using a random-sized, normalized kernel within specified limits, containing $1\text {s}$ in a randomly drawn line and $0\text {s}$ otherwise. This describes an effect that usually results from camera motion during an exposure window.
- *HueSaturationValue* This augmentation randomly changes hue, saturation and value (HSV) of an image within the specified limits.

**Figure 3:**
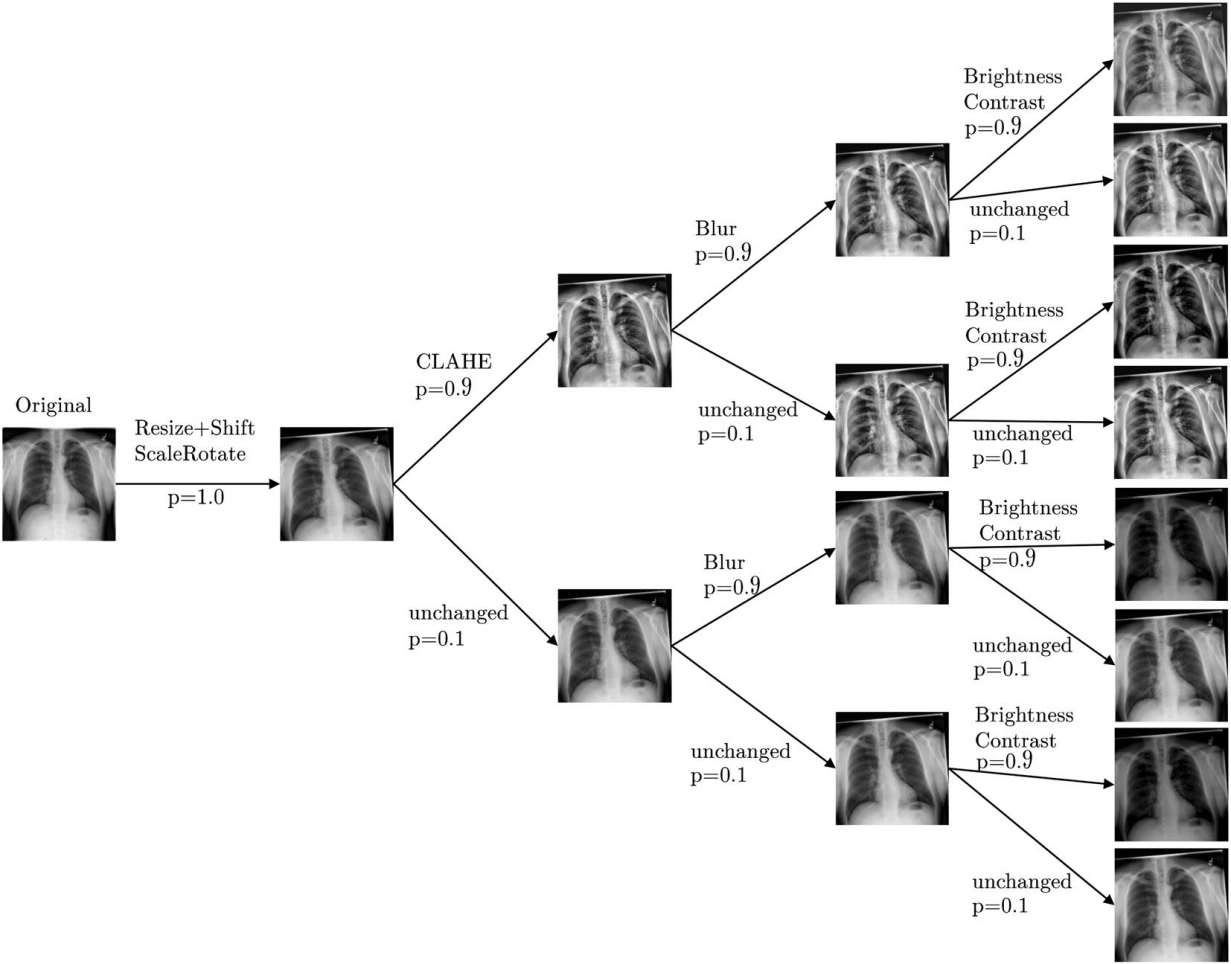
Stacking of probabilistic transformations in a pipeline can result in many different augmented versions of an image.

The augmentation pipelines apply different transformations in a probabilistic way from top to bottom. This means that each transformation is sequentially only applied with a certain probability and the transformations stack on top of each other. This results in a tree-like structure of transforms and yields many possible augmented versions of an image, as showcased by Fig. 3.

**Figure 4:**
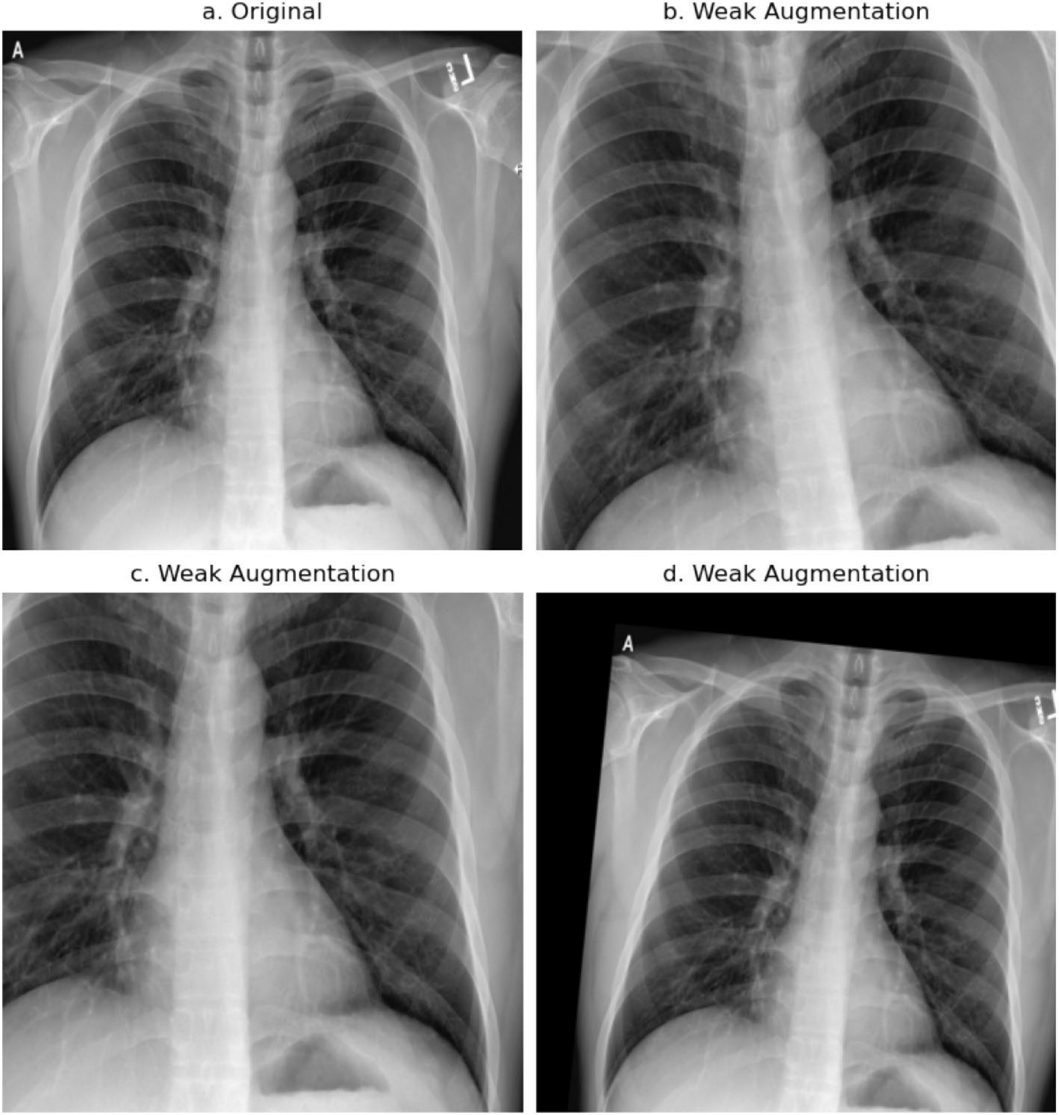
Collection of weak augmentations, only applying affine transforms like shifting, scaling and rotating.

**Figure 5:**
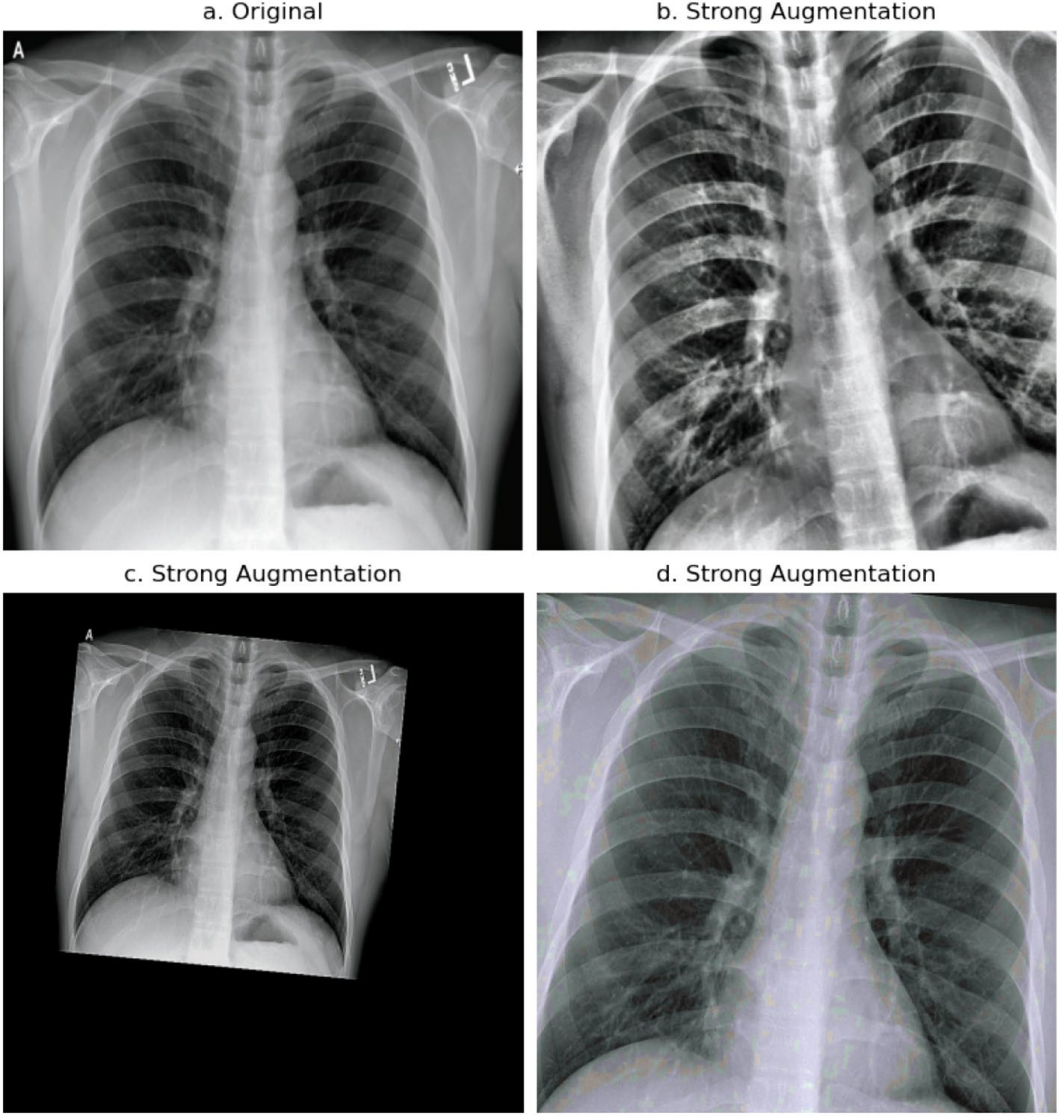
Collection of strong augmentations, applying affine transforms, as well as brightness and sharpen or blur operations.

The proposed pipelines can be described as **strong** augmentation pipelines and **weak** augmentation pipelines. The strong augmentation pipelines utilize a decent amount of different augmentations, like affine transforms, as well as brightness and sharpen or blur operations. This pipeline was inspired by the winning solution to the 2021 SIIM-FISABIO-RSNA Machine Learning COVID-19 Challenge^73^. The weak augmentation pipeline only consists of shifting, scaling and rotating the image and produces mostly realistic looking images. Figure 4 shows some examples of weak augmentations and Fig. 5 shows examples of strong augmentations. Table 3 shows all transformations of the strong and weak augmentation pipelines. Table 4 shows our augmentation strategies and the corresponding augmentation pipeline that is applied to majority and minority classes.

**Table 3:** Strong and weak augmentation pipelines.

Augmentation	Parameters	Probability
Strong augmentations		
Resize	height=224, width=224	1.0
ShiftScaleRotate	scale_limit=0.5, rotate_limit=10, shift_limit=0.1	1.0
One of:		0.9
[CLAHE, RandomBrightnessContrast, RandomGamma]	clip_limit=4.0, grid_size=(8,8)	1.0
	brightness_limit=0.2, contrast_limit=0.2, brightness_by_max=True	1.0
	gamma_limit=(80,120)	1.0
One of:		0.9
[Sharpen, Blur, MotionBlur]	alpha=(0.2,0.5), lightness=(0.5,1.0)	1.0
	blur_limit=7	1.0
	blur_limit=7	1.0
One of:		0.9
[RandomBrightnessContrast, HueSaturationValue] Normalize	brightness_limit=0.2, contrast_limit=0.2, brightness_by_max=True	1.0
	hue_shift_limit=20, sat_shift_limit=30, val_shift_limit=20	1.0
	mean=(0.485, 0.456, 0.406), std=(0.229, 0.224, 0.225)	1.0
Weak augmentations		
Resize	height=224, width=224	1.0
ShiftScaleRotate	scale_limit=0.5, rotate_limit=10, shift_limit=0.1	1.0
Normalize	mean=(0.485, 0.456, 0.406), std=(0.229, 0.224, 0.225)	1.0

Augmentations carried out with Albumentations library^71^.

**Table 4:** Augmentation strategies with their respective augmentation pipelines.

Augmentation strategy	Majority class (Healthy)	Minority class (Severity classes 1-5)
Weak–weak	Weak augmentations	Weak augmentations
Strong–weak	Strong augmentations	Weak augmentations
Strong–strong	Strong augmentations (without ShiftScaleRotate)	Strong augmentations
Weak–strong	Weak augmentations	Strong augmentations

#### Baseline model

This model does not use ROS during training and therefore acts as a baseline model to facilitate a benchmark for oversampling strategies. It utilizes the weak augmentation pipeline, consisting of affine transforms to serve as a baseline. The same augmentations are applied to all classes.

#### Weak–weak augmentation strategy

In this strategy, instances of the minority classes use ROS during training. Each sample is weighted with its inverse class weight. This leads to a uniform distribution over all classes for the model to train with. The samples are not modified any further and the weak augmentation pipeline is used for all classes, regardless of occurrence. This strategy largely resembles ROS and can serve as a point of reference.

#### Strong–weak augmentation strategy

This augmentation strategy uses strong augmentations for the majority class and weak augmentations for the minority class. The idea is to intentionally reduce the image variations of the minority class and provide largely reasonable X-ray images. This enables the model to train with images that are more closely related to the image space of the validation images, where no augmentation is present. This reduces the shift between train and validation data and could therefore improve classification of underrepresented classes.

#### Strong–strong augmentation strategy

This augmentation strategy uses strong augmentations for the majority class as well as for the minority classes. We introduce a small difference between majority and minority classes by removing the shifting, scaling and rotating augmentations for the majority class. The idea is to use extensive augmentations for all classes, while still providing extra image variations to the minority classes. This could lead to an all around robust model with more realistic image variants for the minority classes.

#### Weak–strong augmentation strategy

This augmentation strategy uses weak augmentations for the majority class and strong augmentations for the minority class, reversing the augmentation layout of the strong–weak strategy. This increases variants in the image space for minority classes during oversampling, while keeping the majority class largely as is. The large increase in image variants mimics synthetic creation, for example through interpolation^11,12^ or GAN-based approaches^51,74–76^. Since the majority class is often not augmented in these methods, we use only weak augmentations to produce realistic looking images.

### Ethical approval

The ethics board of the Medical Faculty and the University Hospital in Ulm approved this retrospective evaluation study and waived the informed consent requirement (No. 271/20).

## Results

We evaluate our augmentation strategies with a ConvNeXt-S model, employing each strategy during training. The resulting models are evaluated based on precision, recall, F1-score, accuracy, receiver operating characteristic (ROC) curves and the area under the ROC curve (AUC). A holdout-validation would be unfeasible due to the low amount of data in the minority classes. Therefore, we base our evaluation on a 5-fold cross-validation. The mean ± standard deviation values are calculated based on the respective validation split of each fold.

One of the challenges with evaluation is to show the effect of class imbalance on the performance of our models. Since some metrics are sensitive to class imbalance and some are not, we can illustrate the effect of our augmentation pipelines in this imbalanced learning scenario. This is the main reason we include the accuracy, although we do not regard it as the primary performance metric for this imbalanced problem and even see it as misleading. Still, we use it as a reference to emphasize the discrepancy to more adequate metrics that are insensitive to class imbalance. In the following we look at single class and aggregated results separately, because the performance for the most severe cases is more important than overall results in this medical setting.

### Single class results

Table 5 shows precision, recall, F1-score and AUC for each class independently for all augmentation strategies. Examining the performance on the most severe, and therefore least frequent, classes is of medical relevance and arguably more important than overall model performance.

**Table 5:** Evaluation metrics for each class independently for all augmentation strategies.

	Precision	Recall	F1-score	AUC
Baseline (no oversampling)				
Healthy	.9806 ± .0021	$.9861 \pm .0044$	**.9833** ± **.0016**	**.9832** ± **.0049**
Severity 1	**.6741** ± **.0406**	.5088 ± .0537	$.5771 \pm .0322$	$.9541 \pm .0120$
Severity 2	$.6257 \pm .0385$	**.7397** ± **.0376**	**.6761** ± **.0165**	**.9717** ± **.0023**
Severity 3	**.5894** ± **.0442**	.4655 ± .1420	$.5030 \pm .0887$	**.9761** ± **.0037**
Severity 4	$.2083 \pm .1143$	$.0819 \pm .0520$	$.1137 \pm .0661$	**.9730** ± **.0153**
Severity 5	$.1000 \pm .2000$	$.0667 \pm .1333$	$.0800 \pm .1600$	$.9816 \pm .0281$
Weak–weak strategy				
Healthy	**.9814** ± **.0042**	$.9742 \pm .0085$	.9777 ± .0027	$.9826 \pm .0045$
Severity 1	$.5323 \pm .0759$	**.6804** ± **.0637**	**.5901** ± **.0395**	$.9576 \pm .0111$
Severity 2	$.6446 \pm .0399$	.5677 ± .0511	.6003 ± .0152	$.9634 \pm .0060$
Severity 3	$.5168 \pm .0220$	**.5763** ± **.0857**	**.5434** ± **.0499**	$.9725 \pm .0047$
Severity 4	$.2018 \pm .0596$	$.1895 \pm .0661$	$.1873 \pm .0567$	$.9308 \pm .0498$
Severity 5	**.4000** ± **.3432**	$.2333 \pm .1225$	$.2667 \pm .1520$	$.9617 \pm .0354$
Strong–weak strategy				
Healthy	$.9416 \pm .0050$	$.9713 \pm .0133$	$.9561 \pm .0043$	$.9464 \pm .0109$
Severity 1	$.4264 \pm .0571$	$.4170 \pm .0747$	$.4150 \pm .0344$	$.9231 \pm .0193$
Severity 2	$.6253 \pm .0693$	$.3712 \pm .0699$	$.4574 \pm .0400$	$.9397 \pm .0100$
Severity 3	$.5279 \pm .0509$	$.4496 \pm .1008$	$.4769 \pm .0656$	$.9571 \pm .0071$
Severity 4	$.2522 \pm .1382$	**.3552** ± **.1578**	**.2662** ± **.1148**	$.9412 \pm .0277$
Severity 5	$.2567 \pm .1638$	$.3000 \pm .1633$	$.2671 \pm .1465$	$.9755 \pm .0262$
Strong–strong strategy				
Healthy	$.9350 \pm .0113$	**.9890** ± **.0030**	$.9612 \pm .0064$	$.9736 \pm .0125$
Severity 1	$.4821 \pm .0679$	$.3904 \pm .0305$	$.4275 \pm .0233$	$.9474 \pm .0161$
Severity 2	$.6624 \pm .0520$	$.3197 \pm .1102$	$.4185 \pm .0986$	$.9584 \pm .0070$
Severity 3	$.5131 \pm .0828$	$.3833 \pm .1098$	$.4250 \pm .0641$	$.9635 \pm .0105$
Severity 4	**.2830** ± **.0694**	$.2181 \pm .0742$	$.2403 \pm .0556$	$.9684 \pm .0115$
Severity 5	.3800 ± .1939	**.3333** ± **.1826**	**.3489** ± **.1783**	$.9793 \pm .0278$
Weak–strong strategy				
Healthy	$.9721 \pm .0057$	$.9851 \pm .0023$	$.9785 \pm .0027$	$.9817 \pm .0021$
Severity 1	$.5628 \pm .0392$	$.6203 \pm .0895$	$.5859 \pm .0407$	**.9621** ± **.0067**
Severity 2	**.6887** ± **.0521**	$.5406 \pm .0685$	$.6003 \pm .0254$	$.9687 \pm .0035$
Severity 3	$.5128 \pm .0583$	$.5059 \pm .0695$	$.5070 \pm .0534$	$.9731 \pm .0032$
Severity 4	$.2195 \pm .0620$	$.1762 \pm .0693$	$.1913 \pm .0604$	$.9625 \pm .0160$
Severity 5	$.3045 \pm .3669$	$.3167 \pm .2906$	$.2705 \pm .2531$	**.9853** ± **.0162**

Significant values in bold.

Unsurprisingly, the baseline model shows strong performance for more frequent classes, especially for precision and AUC values, although the margin to Weak–weak and Weak–strong is comparatively small. Unfortunately, the baseline model has low precision and recall values for severity 4 and 5, rendering the model unsuitable for these important cases. The Weak–weak model shows good all-around performance and strong recall values for severity 1 and 3, but is quite weak for severity 4 cases. All proposed augmentation strategies improve recall and F1-score values for severity 4 and 5 cases significantly, with Strong–weak showing the best recall and F1-score for severity 4. Strong–strong shows the best precision for severity 4 and best recall and F1-score for severity 5. This suggests, that a model trained with this strategy is therefore best suited to detect the least frequent (and in this study: most severe) cases. The Weak–strong augmentation strategy shows good all around results, but does not excel in any one class.

In conclusion, the baseline and Weak–weak models show predominant performances for majority classes, while the various augmentation strategies excel for minority classes. The proposed augmentation strategies might encapsulate smaller intricacies for these less frequent cases and suggest the use of specialized augmentation pipelines, designed for minority classification. Although the oversampling leads to a reduced performance on the more frequent classes healthy and severity 2, we still see better recall and precision values for the healthy class in the proposed augmentation strategies.

### Aggregated results

Table 6 shows aggregated metrics and overall model performance for all augmentation strategies. Similar to single class performances, we employ different aggregation methods to show the discrepancy between the methods that are sensitive or insensitive to class imbalance. The macro-averages are calculated by taking the unweighted mean over all classes and are therefore insensitive to class imbalance. The weighted averages are calculated by taking the average for each class and weighting by their support, making them sensitive to class imbalance. Looking at the difference between these two values illustrates the significant impact of class imbalance in this study. Micro-averages are not shown, since they equal accuracy. The macro-average AUC is calculated by pairwise comparison between all classes and calculating the average (One-vs-One strategy), which better reflects the statistics of the less frequent classes.

**Table 6:** Aggregated metrics as macro-averages and weighted averages for all augmentation strategies.

	Precision	Recall	F1-score	AUC	Accuracy
Baseline (no oversampling)					
Macro-average	$.5297 \pm .0469$	$.4748 \pm .0309$	$.4889 \pm .0383$	.8762 ± .0097	**.9314** ± **.0048**
Weighted average	.9289 ± .0034	.9314 ± .0048	.9283 ± .0048	-	
Weak–weak strategy					
Macro-average	**.5462** ± **.0610**	**.5369** ± **.0189**	**.5276** ± **.0260**	.8743 ± .0189	$.9191 \pm .0052$
Weighted average	.9241 ± .0023	$.9191 \pm .0052$	$.9204 \pm .0034$	-	
Strong–weak strategy					
Macro-average	$.5050 \pm .0505$	$.4774 \pm .0181$	$.4731 \pm .0361$	$.8530 \pm .0167$	$.8907 \pm .0081$
Weighted average	$.8852 \pm .0056$	$.8907 \pm .0081$	$.8841 \pm .0045$	-	
Strong–strong strategy					
Macro-average	$.5426 \pm .0344$	$.4390 \pm .0293$	$.4702 \pm .0319$	$.8461 \pm .0178$	$.8988 \pm .0130$
Weighted average	$.8839 \pm .0131$	$.8988 \pm .0103$	$.8846 \pm .0145$	-	
Weak–strong strategy					
Macro-average	.5434 ± .0604	$.5241 \pm .0539$	.5222 ± .0450	**.8817** ± **.0192**	$.9224 \pm .0018$
Weighted average	$.9201 \pm .0028$	$.9224 \pm .0018$	$.9199 \pm .0027$	-	

The macro-average recall equals balanced accuracy. Significant values in bold.

Since this study examines an imbalanced class problem, the weighted averages can give a misleading impression of model performance because they underestimate the importance of the less frequent and severe cases. We therefore assess the model performance primarily on the macro-averages and keep the weighted averages only as an indication of discrepancy. The averaged results show strong performances mostly for the Weak–weak and Weak–strong strategy. While Weak–weak exhibits the best performance on precision, recall and F1-score, Strong–weak shows the highest AUC value. Surprisingly, the Weak–strong model shows the best values for AUC. This was already hinted at in Table 5, where the model shows good all-around performances and the best AUC values for Severity 1 and 5. This demonstrates, that single class investigations might be preferred over aggregated results in the context of imbalanced learning with important minority classes. These results suggest the use of either the Weak–weak or Weak–strong model for the presented use case.

**Figure 6:**
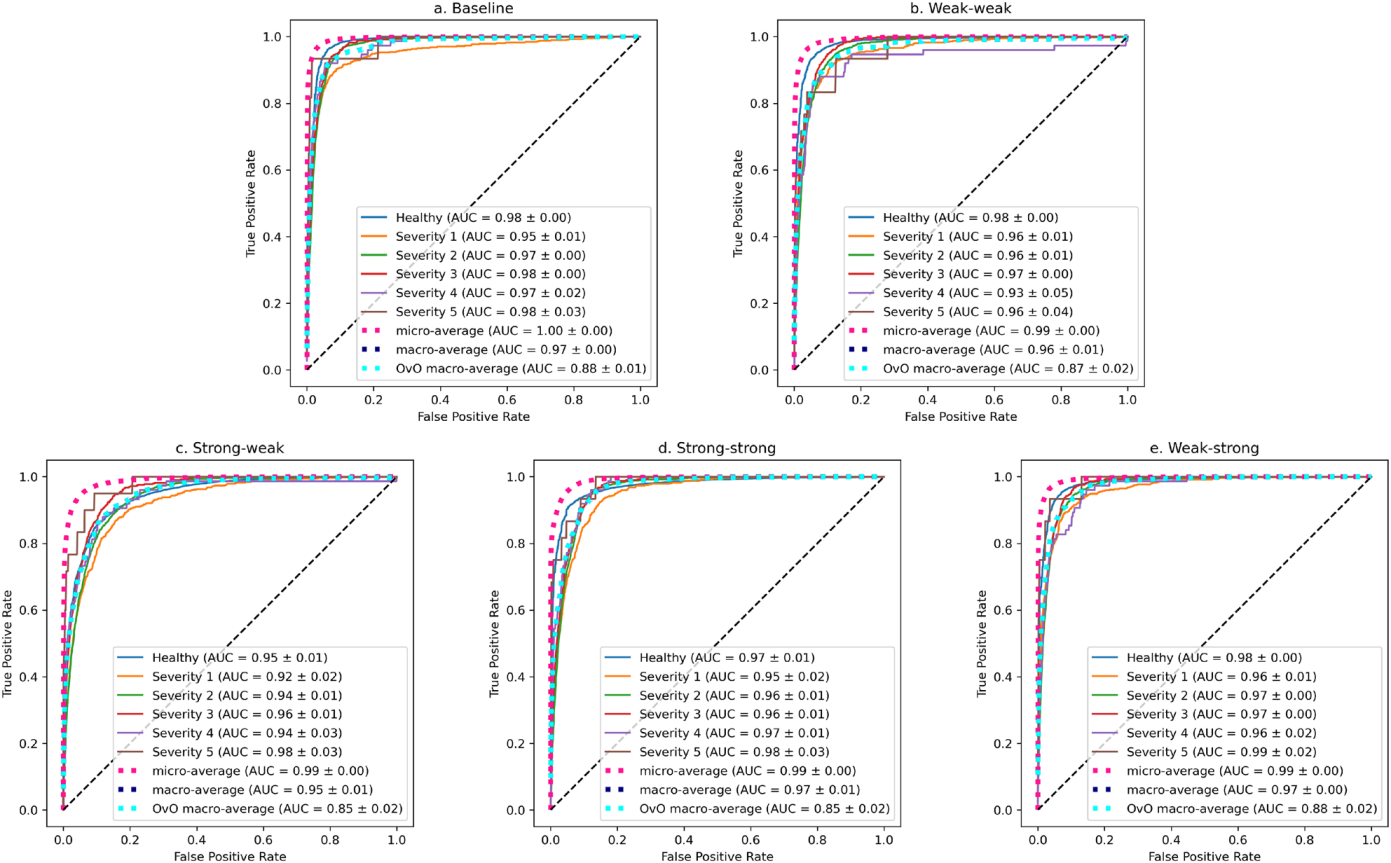
ROC curves for the baseline model (**a**), and all augmentation pipelines (**b**–**e**).

Figure 6 shows the average ROC curves across all folds of the 5-fold cross-validation. ROC curves for single classes are computed with the One-vs-Rest strategy, regarding the remaining classes as the negative class as a bulk. This strategy is sensitive to class imbalance, because the negative group can be affected by class imbalance, even for macro-averages. To alleviate this effect, we also calculate the OvO macro-average with the One-vs-One strategy by calculating average curves from pairwise comparison of all classes. The micro-average is calculated globally over all samples and is therefore sensitive to class imbalance, which can give a misleading impression about performance in our problem and does not convey much information. The macro-average is calculated independently for each class and then averaged, treating each class equally regardless of distribution.

The baseline and Weak–strong models show the best ROC curves. This is not very surprising in the case of the baseline model, since ROC curves are sensitive to class imbalance. They show the best OvO macro-average curve, followed by the Weak–weak strategy. In conclusion, the baseline and Weak–strong models show very similar ROC curves, while Weak–weak, Strong–strong, and Strong–weak models are slightly worse.

### Explainability

**Figure 7:**
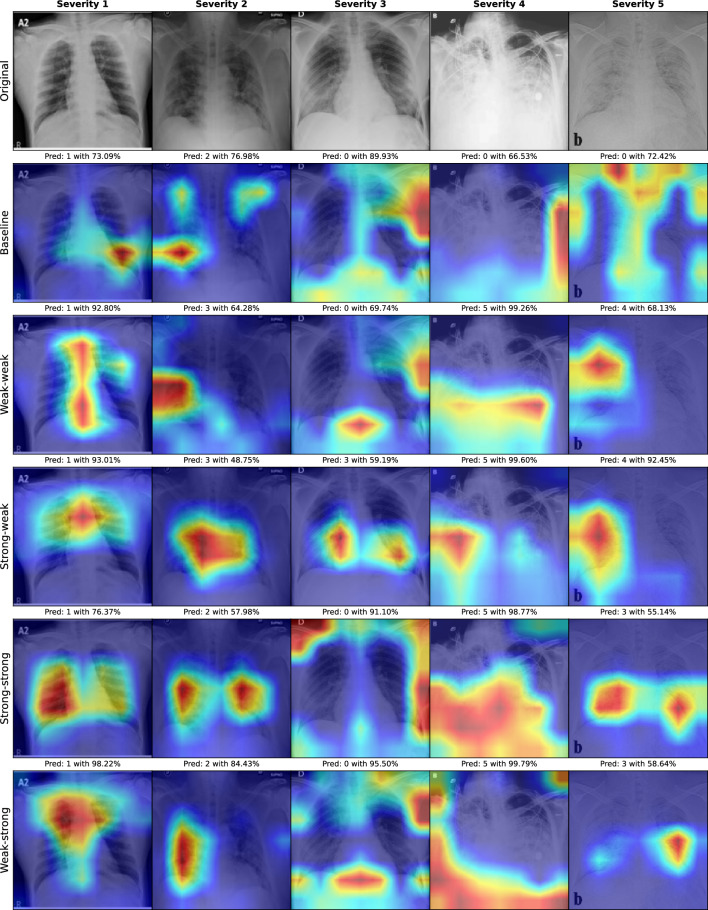
GradCAM attributions for sample images from severity 1–5 and all proposed augmentation strategies. Predicted severity with prediction score on top. A prediction of 0 indicates the healthy class.

To further explore differences in important classification areas for our strategies, we provide GradCAM^77^ attributions. GradCAM is a method to visualize gradients of the classification score with respect to the final convolutional feature map and therefore highlights significant regions of an image. Figure 7 shows the GradCAM attributions for sample images with severity 1–5 and all proposed augmentation strategies. To ensure a consistent comparison, attributions have been calculated for models with the same validation fold. Therefore, none of the demonstrated samples were part of the training data for these models.

The GradCAM attributions entail some interesting findings. First of all, the baseline model has trouble identifying the severity cases 3–5 and highlights mostly areas outside the lungs, while the severity 3 image seems to be problematic for all models except Strong–weak. Secondly, the highlighted areas can differ a lot between strategies, even for the same image and for consistent classifications. This could be an indication for the high variance that is introduced during a training process with limited amounts of data and further reinforces the necessity of an oversampling strategy in such scenarios. Thirdly, severity 4 and 5 images are classified as severe by our proposed models (although in reverse order) with infiltrated lung areas highlighted. This is not the case for the baseline model, attributing mostly unaffected areas outside the lung.

## Limitations and discussion

In this work, we provided severity scores for all COVID-19 positive images in the COVIDx8B CXR data collection, making it one of the largest COVID-19 severity databases for CXR images. Severity scores are important to quickly detect the most severe cases in an emergency scenario and act appropriately. Furthermore, we trained and evaluated deep learning models on the severity dataset to provide a benchmark for the automated severity classification task. Since the most severe cases are the least frequent, this skewed dataset complicates the training process for deep learning models and is detrimental to performance, especially on the important minority classes. To alleviate this problem and improve classification performances, we proposed multiple augmentation strategies, consisting of different augmentation pipelines for majority and minority classes with an oversampling strategy. We cross-validated these strategies based on appropriate metrics for imbalanced learning problems. Our augmentation strategies show significant improvements in precision and recall values for the rare and most severe cases, while achieving robust performances overall.

Our results show that classification metrics for more frequent classes can be improved by using weak augmentations, while the performance on rare classes seem to favor stronger augmentations. Learning robust representations for classes with a very low amount of samples is non-trivial and usually introduces larger generalization gaps between training and testing data^5^. While weak augmentations seem to be adequate to learn representations for more frequent classes, they do not sufficiently reduce overfitting for less frequent classes. For these cases, stronger augmentations introduce more noise to the underrepresented classes and help to reduce model variance and potential overfitting problems. This dependency between the amount of noise introduced by stronger augmentations and the scarcity of data should be researched more rigorously in future works.

We notice that the impact on performance of our augmentations can vary across different classes. While we are not entirely sure why this is the case, we suspect that performance on different classes could benefit from more specific augmentations. This makes sense intuitively, since different classes occupy different image spaces, where some augmentations can be more sensible than others. After all, the goal of augmentations is to increase density of the image space, without leaving the classes subspace. While most research focuses on augmenting the minority class only^51,74–76^, the idea of utilizing class-specific image augmentations could be a promising research direction. This notion shares some similarity with cross-class augmentation strategies based on image-to-image translation^78^, in which images from one class are modified to represent another class.

Although the strategies show improved minority classification, we are aware that these performances might not be enough to fulfill medical requirements. The idea conveyed in this study needs to be further improved upon to warrant clinical use, especially regarding low recall values for the most severe cases. Additionally, although the data was reviewed and labeled by a dedicated thoracic radiologist with 9 years of experience in lung imaging, the severity scores could be cross examined by multiple radiologists. Since the dataset is publicly available, the possibility for comprehensive external validation as well as model benchmarks are given.

However, we are convinced that our investigations represent a good point of reference for further research. In particular, a larger pool of data could also increase model performance significantly, especially for the minority classes. This study only represents the first steps with the dataset provided and opens future opportunities for researchers to explore. It is also worth mentioning, that our AI approach is not limited to COVID-19 and could potentially be used for different lung diseases and types of pneumonia in general, since they exhibit similar infiltration patterns and ground-glass opacities. Future improvements on the dataset could entail the detailed annotation of infiltration in different lung areas, similar to Signoroni et al.^43^. This could enable the training of segmentation models and yield further information on affected lung regions, linking severity to the infected lung volume.

The augmentation pipelines proposed in this work proved to function well in practice^73^, but they are manually designed and might not work well for different applications. Automatic generation of augmentation pipelines like AutoAugment^79,80^, RandAugment^81^ or TrivialAugment^82^ could therefore be interesting approaches to combine with our imbalance-specific augmentation strategies. This could also enable class-specific image augmentations, since designing them manually might be infeasible.

Although the GradCAM attributions provided some insight on the differences between our proposed strategies, they are themselves noisy and show lots of variance between the models. This could be improved by aggregating and smoothing attributions over many images or by evaluating the quality of the attributions with respect to the classification results^83,84^.

## Data Availability

The severity labels and code is publicly available under hhttps://github.com/dschaudt42/covid-severity-aug. The original COVIDx8B dataset is available from https://github.com/lindawangg/COVID-Net.
